# Real-world evidence: experiences and challenges for decision making in Latin America

**DOI:** 10.1017/S0266462323002647

**Published:** 2023-12-18

**Authors:** Sebastián García Martí, Andrés Pichón-Riviere, Federico Augustovski, Manuel Espinoza

**Affiliations:** 1 Institute for Clinical Effectiveness and Health Policy (IECS‐CONICET), Buenos Aires, Argentina; 2 Department of Public Health, School of Medicine, Pontificia Universidad Católica de Chile, Santiago, Chile

**Keywords:** real-world data, real-world evidence, technology assessment, Latin America, coverage

## Abstract

**Objective:**

The Health Technology Assessment (HTA) process aims to optimize health system funding of technologies. In recent years there has been an increase in what is known as Real-World Evidence (RWE) as a complement to clinical trials. The objective of Health Technology Assessment International’s Latin American Policy Forum 2022 was to explore the utility of incorporating RWE into HTA and decision-making processes in the region.

**Methods:**

This article is based on a background document, survey, and the deliberative work of the country representatives who participated in the Forum.

**Results:**

There is a growing interest in the use of Real-World Data / Real-World Evidence in HTA processes in Latin America, although currently there are no specific local guidelines for RWE use by HTA agencies. At present, its use is limited to certain areas such as adding context to HTA reports, the evaluation of adverse events, or cost estimation.

Potential future uses of RWE were identified, including the creation of risk-sharing agreements, the assessment of technology performance in routine practice, providing information on outcomes that are not so easily evaluated in clinical trials (e.g., the identification of specific subpopulations or quality of life), and the estimation of input parameters for economic evaluations.

**Conclusions:**

The participants agreed that there are several areas presenting significant potential to expand the application of RWD/RWE and that the development of normative frameworks for its use could be helpful.

## Introduction

Health Technology Assessment (HTA) is a multidisciplinary process that uses explicit methodologies to determine the value of a health technology throughout its life cycle (1). Its purpose is to inform the decision-making process on technology funding and coverage to promote equitable, efficient, and high-quality health systems. This information is used by health systems to make decisions that affect the allocation of health resources and people’s access to health technologies and services.

Real-world evidence (RWE) has gained significant traction in recent years due to its potential to provide valuable insights into the real-world effectiveness, safety, and cost-effectiveness of health technologies. According to the glossary of Health Technology Assessment International (HTAi), RWE is defined as “evidence derived from the analysis of real-world data, which includes data collected for purposes other than specific research that provides information about the routine delivery of health care and the health status of the target population” (2).

While it is true that RWE is considered of potential use for HTA, reimbursement, and pricing, it is important to note that RWE is not always accepted nor is the evidence considered at the same level by different countries (3). While RCTs (randomized controlled trials) are still considered the gold standard for demonstrating efficacy, RWE can complement and provide additional insights beyond what controlled trials can offer. RWE can provide real-world effectiveness data, assess long-term outcomes, evaluate safety in larger populations, and inform on the value and cost-effectiveness of health technologies in real-world settings.

The incorporation of RWE studies in different phases of the HTA process has been recognized as potentially useful in certain situations where clinical trials face limitations. RWE can help characterize patients with unmet needs or diseases that are poorly controlled, identify infrequent adverse events through pharmacovigilance, and describe the usual patterns of treatment that allow for the identification of standard comparators in a clinical trial or economic evaluation. Additionally, RWE can provide information for the execution of risk-sharing agreements or the monitoring of interventions once they are incorporated. However, it is important to acknowledge that RCTs remain the gold standard for demonstrating efficacy and are often required for regulatory approval (4). Moreover, RWE has implications beyond HTA, being recognized in earlier regulatory stages as well as in post-reimbursement monitoring (5).

Since 2016, HTAi has organized the Latin American Policy Forum (Policy Forum), which has the objective of providing a neutral space for strategic discussions about the current state of HTA, its development, and implications for the health system, industry, patients, and other stakeholders (6; 7).

The recent seventh Latin American Policy Forum focused on exploring the usefulness of incorporating RWE studies into HTA and decision-making processes. It aimed to analyze the problems faced by HTA agencies and other stakeholders regarding the use of RWE and define a set of key principles and actions to guide its utilization and development in Latin America. Although this article does not represent a formal consensus document and should not be interpreted as representative of the participants’ points of view, it summarizes the discussion during the Policy Forum, providing valuable insights into the challenges and opportunities associated with RWE utilization in Latin American region.

## Methods

The scientific secretariat prepared a background document summarizing the potential uses of RWE, as well as the barriers and challenges to its incorporation into HTA that face countries around the world (8). This article was informed by scientific and gray literature collected through an unstructured search based on recent key publications and discussions held with members of the Forum organizing committee. Documents and websites of HTA agencies and governments were reviewed to identify frameworks for RWE use (United Kingdom, Canada, USA). The primary objective of the background document was to provide a general piece of information to the Forum participants, harmonize definitions of key terms, and help to enable discussions during the face-to-face meeting. Also, with the objective of having some basic understanding of the situation in the region, a survey was administered to the representatives of the participating countries of the Policy Forum regarding their current status of incorporating RWE information in HTA processes.

The Policy Forum was held in person on August 15th and 16th, 2022, in Brasilia. There were 45 participants, with a total of 11 countries from the region represented: Argentina, Brazil, Chile, Colombia, Costa Rica, Ecuador, El Salvador, Mexico, Paraguay, Peru, and Uruguay. The main HTA agencies in the region were represented. Annex I of the Supplementary Material contains the list of participants along with their affiliations and countries. The meeting format included keynote presentations, breakout group work, and plenary discussions.

The aim of the first day was to introduce, from international and regional perspectives, the topic of RWE use and its incorporation into HTA processes from the viewpoint of different stakeholders. A general international perspective was described, drawn from the results of the Global Policy Forum related to RWE (9), along with an analysis of the challenges and experiences of different countries around the world. A specific example from the United Kingdom was looked at, describing their process to develop a normative framework for the incorporation of RWE in health technology assessment processes. A selection of countries in the region presented their current situation regarding the use of RWE. Additionally, the perspectives of a representative of the drug and device industry and those of a patient representative were shared.

Also, a series of breakout group activities were carried out based on a discussion and debate methodology, with practical processes to redefine problems and try to find solutions (10; 11). Participants were divided into breakout groups that were balanced in terms of the countries and stakeholders represented. After each breakout group activity, the group results were presented and discussed in plenary sessions.

The first breakout group activity was to describe the current situation in the region in relation to the use of RWE. Potential advantages and disadvantages of using RWE in the HTA process were first identified from the perspective of different stakeholders (patients/users, funders, technology producers, HTA agencies) followed by the identification of possible priority uses of RWE in HTA processes in the region through a voting methodology supported by a virtual platform.

The second breakout group activity consisted of identifying barriers and facilitators for the appropriate use of RWD/RWE in HTA processes in the region. This was done via discussions both in the breakout groups and in a subsequent plenary session, supported by a computerized voting system. In addition, the breakout group activity and subsequent plenary argued and discussed a series of potential recommendations and future actions for HTA agencies in Latin America.

The Forum was conducted under Chatam House Rules (12), and all materials were produced in Spanish and English.

## Results

There was a total of 45 participants in the Forum: 10 representatives of HTA agencies, 5 representatives of public, social security and private funders; 16 industry representatives (pharmaceuticals, medical devices, and diagnostics); 1 representative of the Pan American Health Organization, 2 representatives of patient associations, 5 representatives of HTAi and 6 academics; along with organizers and members of the scientific secretariat for the event.

### Current situation in the region

In advance of the Forum, a survey was administered to the 11 country representatives who are members of the Forum about the use of RWE.

Eighty percent of the countries indicated they do not have specific guidelines for the use of RWE, although two-thirds reported using RWE in the absence of specific guidelines. The most frequent uses mentioned were to provide context for HTA reports, assess adverse events, and assess costs.

Most respondents reported that, over the last 3 years, there has been an increasing demand for using RWE in their countries. In half of the cases, RWE was reported as having been used to assess comparative effectiveness and that high-cost diseases, orphan diseases, procedures, and devices are the most widely targeted pathologies or technologies for the application of RWE.

The table in Annex II of the Supplementary Material details the results of the survey.

### Keynote lectures and stakeholder presentations

The key messages of the two keynote lectures held during the first day to facilitate the use of RWE were as follows:

- Promoting the production of guides for the correct use of RWE throughout the HTA cycle and phases.
- Developing common data models to promote quality improvement and interoperability.
- Building capacity to improve the understanding of RWE and its uses.

During the countries’ presentations, it was highlighted that, in general, RWE is currently used for monitoring technologies that are already reimbursed.

Presentations were also made by patients and technology manufacturers, with the former promoting more active participation in the HTA processes.

### Results of breakout group activities

During the first group activity, the focus was on the identification of potential advantages and disadvantages and potential priority uses of RWE in the region.

Presented below are the results produced from the perspective of each of the stakeholders involved. As can be observed, there were many points in common across the different stakeholders. All participants responded to the best of their knowledge and understanding of the advantages and disadvantages following the perspectives surveyed.

#### Patient perspective

On the positive side, RWE can increase knowledge about diseases, their burden, quality of life, and satisfaction with treatment. It can also lead to quicker access to interventions, provide more information on treatment outcomes, open new opportunities for cooperation, and expand patient participation in HTA processes. Additionally, RWE can capture information from nontraditional sources, which are currently underutilized, and offer a broadened vision of intervention effects over the entire life cycle of technologies. However, on the negative side, patients may receive less effective or harmful interventions (if RWE can not describe the effect properly in the absence of RCT), lose access to a treatment due to negative RWE-based results, and find it difficult to understand the RWE process or share their data due to privacy concerns.

#### Manufacturer perspective

RWE offers manufacturers a range of advantages, such as expanding access to data sources, identifying unmet needs, generating information at a lower cost, confirming long-term effectiveness and safety, faster listing and reimbursement processes, potential to expand indications, promoting earlier access to technologies, and promoting greater interaction between stakeholders. However, there are also some disadvantages, including unclear rules on how to use RWE, increased potential for bias, reduced sales of certain products, potential for off-label use, and discrepancies between real-world results and clinical trials.

#### Funder perspective

Advantages include increased agreement between stakeholders, better information to inform decision making, understanding the effects of interventions in practice, knowledge of the return on investment, and greater value for populations traditionally excluded from RCTs. Disadvantages include a potential increase in coverage and financial burden, decision making in uncertain situations, and higher costs for data collection and sharing.

#### HTA agency perspective

RWE has the potential to provide powerful insights to HTA agencies, but comes with both advantages and disadvantages. Advantages include the ability to bring together data from various sources, understand outcomes/effectiveness of treatments where RCTs are not available (e.g., rare diseases), obtain data that cannot be obtained in RCTs, supplement RCTs from other regions with local data, generate information through data that is more similar to the target population, and more data to enable better disease modeling in economic evaluations studies. Disadvantages include difficulty in accessing good-quality data, more complex studies, heterogeneity of variable quality, increased demand for studies and analysis, uncertainty in the results, and particular regulatory aspects or lack of regulation.

#### Provider perspective

From a provider perspective, RWE can be both beneficial and detrimental. Advantages include the promotion of the collection of information about usual practice, as well as the ability to better understand the results of this practice. However, there are also potential risks associated with the use of real-world evidence, such as the use of interventions that may be ineffective or even potentially harmful, as well as an increased administrative burden.

**Figure 1.**
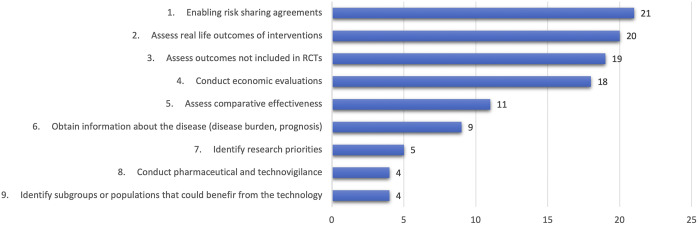
Potential uses of RWE in the Latin American Region.

Also as part of this group activity, a list of the main potential uses that RWE could have in the region was produced (Figure 1). These were initially identified in the breakout group activities and subsequently prioritized by all Forum participants through an electronic vote. The three most voted potential uses were the utilization of RWE in risk-sharing agreements (21 votes), generating information about real-life utilization of technologies (20 votes), and exploring outcomes not included in RCTs (19 votes).

The objective of the second group’s activities was to identify barriers/facilitators for the correct use of RWD/RWE in HTA in the region (Tables 1 and 2).

**Table 1.** Main barriers identified for the correct use of RWE in the region

Lack of trust and acceptance by stakeholders. Lack of a regulatory framework that identifies, e.g., the types of questions that can be answered or the role of RWE in the HTA process. Lack of a data governance system. Ethical and legal aspects (both in data quality and confidentiality). Lack of in-depth knowledge on the subject by different stakeholders (both at the individual and institutional levels, e.g., regulatory agencies accustomed only to RCTs). Lack of methodological manuals. Lack of training and human resources. Lack of resources to implement RWD/RWE. Low level of engagement by providers. Restricted data access. Challenges to the transferability of RWD across different settings or countries. Lack of infrastructure and interoperability of data sources.

**Table 2.** Main facilitators identified for the correct use of RWE in the region

Interest and political will of stakeholders on the issue (although perhaps only among a small group of interested parties). Willingness to establish regional synergies. Existence of international guidelines and standards. Declarations of relevant organizations that facilitate acceptability. Existence of public-private initiatives addressing concrete problems. Availability of good data at the individual and aggregate-levels (although it can be heterogeneous between countries). Increasing availability of public databases. Post-Covid-19 aspects and current economic restrictions increasing pressure to apply new methodologies and accelerate data collection. Existence of data protection legislation. Increasing technical capacity to analyze and aggregate data. Methodologies such as artificial intelligence and machine learning facilitate the extraction and analysis of large data sets. Fertile ground for advancing risk sharing agreements.

**Figure 2.**
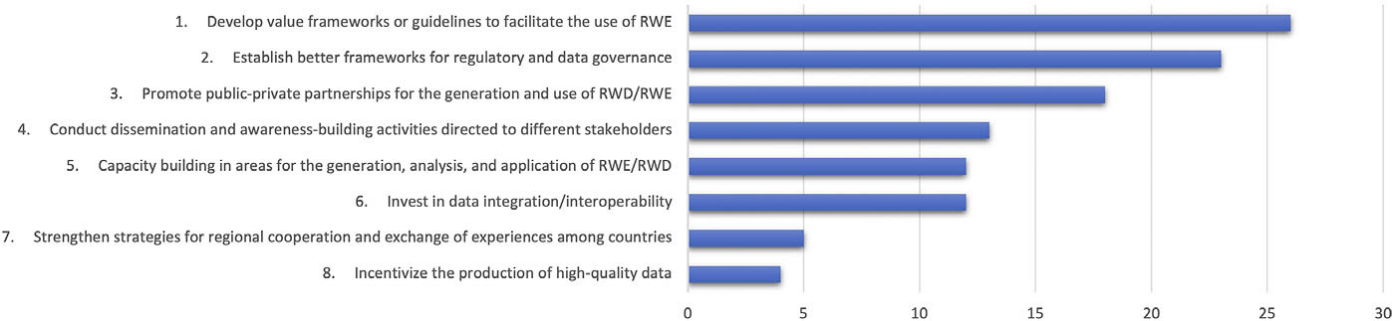
List of recommendations to promote RWE utilization in the Latin American Region.

Also, a list of recommendations to advance the correct use of RWE in the region was developed. The recommendations were first described by the groups and then prioritized in a plenary session by all Forum participants through an electronic vote. Figure 2 presents the identified strategies.

Additionally, there also arose some initiative to develop a framework document for the correct use of RWE in the region that could be jointly promoted by the different countries and in collaboration with organizations such as the Panamerican Health Organization (PAHO) and HTAi.

## Conclusions

The 2022 Policy Forum on the use of RWE showed there is a growing interest in this topic in the region, given that RWE can complement the evidence from controlled clinical trials to answer many relevant HTA questions. In the pre-Forum survey that provided an overview of the current situation of RWE use in the region, most of the countries mentioned they do not have specific guidelines for RWE use, although more than half of them report using it for different situations, among which include providing context in HTA reports, assessing adverse events, and estimating costs. The use of RWE for estimating comparative effectiveness is not yet widely applied. These findings are similar to other studies carried out in the region (13, 14). With regard to the potential advantages or disadvantages of RWE use in the region, in general, there was some agreement about its value in promoting earlier access to technologies, although the difficulties in conducting this type of study were pointed out, both in a methodological sense and regarding human resources. These studies present a greater degree of uncertainty regarding their results, and there is a lack of decision-maker confidence in them, either due to a greater risk of bias or difficulties in accessing good-quality data. Among the priority applications or uses that RWE could have in the region was the creation of risk-sharing agreements, assessing the performance of technologies in routine practice, providing information on outcomes that are not so easily assessed in RCTs (e.g., the identification of specific subpopulations, longer-term follow-up, or aspects related to patient quality of life), and the estimation of input parameters for economic evaluations.

Some of the potential challenges to the advancement in RWE production and use in the region were a lack of confidence on the part of the different stakeholders to produce and share RWD; the absence of appropriate regulatory frameworks and data governance systems at the country level; poor access to good-quality data; the need for more skilled human resources for the production, analysis, and application of RWD/RWE; low levels of provider engagement and lack of appropriate incentives to improve the quantity and quality of data collection (for example the development of anonymized repositories of data that can be used to generate RWE), and deficiencies in data infrastructure and interoperability.

The challenges faced in Latin America including the need for increased stakeholder confidence in producing and sharing Real-World Data (RWD), the absence of regulatory frameworks and data governance systems as well as issues related to data quality and infrastructure, mirror concerns raised in other regions. The shortage of skilled human resources for RWD analysis and a lack of incentives for data collection are shared challenges that require attention and investment on a global scale (15). Collaboration and knowledge sharing between Latin America and other regions can contribute to advancing RWE’s role in improving healthcare outcomes and access to innovative technologies worldwide.

The Forum also included a discussion of possible actions and recommendations that could promote wider and better use of RWD/RWE in the region. These are as follows:

- Promote the development of a general framework document at the regional level for the correct use of RWE, in collaboration with organizations such as PAHO and HTAi.
- Promote and produce guidelines for the RWE use at the country level (both methodological aspects and their application).
- Advance the regulation and governance of data access (both in the promotion of standards that facilitate interoperability and transferability between different areas or countries, and in increasing their quality).
- Promote public-private partnerships for the production of specific applications of RWD and the use of RWD/RWE
- Conduct dissemination and awareness-building activities on the topic of RWE directed to different stakeholders.
- Build capacity in areas for the production, analysis, and use of RWE/RWD in HTA and decision making.
- Create conditions that incentivize the collection and production of good-quality data.

Over the next few years, it is anticipated that real-world evidence will be increasingly incorporated into HTA and decision-making processes around the globe. The Latin American region is currently following this movement; however, some aspects remain to be further developed such as improvements to real-world data quality and access as well as regulatory frameworks for the use of real-world evidence in HTA processes to enable these types of studies to be appropriately incorporated into decision-making processes in the region.

## Supporting information

García Martí et al. supplementary material 1 García Martí et al. supplementary material

García Martí et al. supplementary material 2 García Martí et al. supplementary material

## Supplementary material

The supplementary material for this article can be found at http://doi.org/10.1017/S0266462323002647.
