# Supplementary material for: Real-world evidence: experiences and challenges for decision making in Latin America
Source: Int J Technol Assess Health Care. 2023 Dec 18;39(1):e73. doi: 10.1017/S0266462323002647 (PMC11579697; doi:10.1017/S0266462323002647)
Supplement: García Martí et al. supplementary material 1 — García Martí et al. supplementary material [file S0266462323002647sup001.docx]

**Annex I**

**Participant List**

| **Name** | **Institution** | **Country** |
| --- | --- | --- |
| Abayubá Perna | Fondo Nacional de Recursos (FNR) | URUGUAY |
| Rob Abbott | HTAi - Health Technology Assessment | CANADA |
| Adriana María Robayo García | Instituto de Evaluación Tecnológica en Salud (IETS) | COLOMBIA |
| Alejandro Araya Andrade | Federación chilena de Enfermedades Raras - FECHER | CHILE |
| Alexandre Lemgruber | Health Technologies PAHO/WHO, OPS/OMS, Regional Advisor | UNITED STATES |
| Ali Powers | HTAi - Health Technology Assessment | CANADA |
| Ana Eduviges Sancho Jimenez | Ministry of Health | COSTA RICA |
| Andrea Gimenez | Ministry of Public Health | URUGUAY |
| Andrés Pichon Riviere | Institute for Clinical Effectiveness and Health Policy (IECS) | ARGENTINA |
| Arely Lemus Carmona | BRISTOL-MYERS SQUIBB |  |
| Ayax Mateos Benítez | Instituto Mexicano del Seguro Social (IMSS) | MEXICO |
| Breanne Dickhout | HTAi - Health Technology Assessment | CANADA |
| Camila Rufino | JOHNSON & JOHNSON |  |
| Dayane Albuquerque | EDWARDS LIFESCIENCES |  |
| Debora Aligieri | Diabetes e Democracia | BRAZIL |
| Diego Guarin | MERCK & CO | ARGENTINA |
| Dino Sepúlveda Viveros | Ministry of Health – Division of Health Planning Undersecretary of Public Health | CHIILE |
| Elio Asano | JOHNSON & JOHNSON |  |
| Federico Augustovski | Institute for Clinical Effectiveness and Health Policy (IECS) | ARGENTINA |
| Felipe Vera Chandia | Ministry of Health - Undersecretary of Public Health in Pharmaceutical Policy and Health Technology Assessment | CHILE |
| Ferananda Laranjeira | MEDTRONIC | BRAZIL |
| Julian Guilherme | PFIZER LIMITED |  |
| Héctor E. Castro- J | F. HOFFMANN-LA ROCHE AG | COLOMBIA |
| Hugo Marín Piva | Caja Costarricense de Seguro Social (CCSSS) | COSTA RICA |
| Javier Garcia | BRISTOL-MYERS SQUIBB |  |
| Joice Valentim | F. HOFFMANN-LA ROCHE AG | SWITZERLAND |
| Jose Thomaz | BIOMARIN |  |
| Juan Santillana Callirgos | ESSALUD - IETSI | PERU |
| Lizbeth Alexandra Acuña Merchan | Cuenta de Alto Costo | COLOMBIA |
| Luisa Abdala | ELI LILLY |  |
| Manny Papadimitropoulos | ELI LILLY | UNITED STATES |
| Manuel A. Espinoza | President of HTAi’s Latin American Policy Forum | CHILE |
| Manuel Donato | CONETEC – Ministry of Health | ARGENTINA |
| Mohit Jain | BIOMARIN |  |
| Pedro Galvan | Instituto de Investigaciones en Ciencias de la Salud (IICS) | PARAGUAY |
| Rabia Kahveci | HTAi - Health Technology Assessment | UKRAINE |
| Rodriguez Grimalt | Institute for Clinical Effectiveness and Health Policy (IECS) | URUGUAY |
| Seamus Kent | NICE - National Institute for Health and Care Excellence | UNITED KINGDOM |
| Sebastán Garcia Marti | Institute for Clinical Effectiveness and Health Policy (IECS) | ARGENTINA |
| Silvana Kelles | UNIMED | BRAZIL |
| Silvina Beatriz Benchtrit | Ministry of Health GCBA | ARGENTINA |
| Suellen Rodrigues | MERCK & CO |  |
| Vania Cristina Canuto Santos | Ministry of Health | BRAZIL |
| Wiija Oortwijn | HTAi - Health Technology Assessment | THE NETHERLANDS |
| William Dorling | PFIZER LIMITED |  |
