# Supplementary material for: Real-world evidence: experiences and challenges for decision making in Latin America
Source: Int J Technol Assess Health Care. 2023 Dec 18;39(1):e73. doi: 10.1017/S0266462323002647 (PMC11579697; doi:10.1017/S0266462323002647)
Supplement: García Martí et al. supplementary material 2 — García Martí et al. supplementary material [file S0266462323002647sup002.docx]

**Annex II**

**Current situation in the region – Survey results**

| **Country** | **Uruguay** | **Chile** | **Paraguay** | **Costa Rica** | **Chile** | **Mexico** | **Argentina** | **Colombia** | **Brazil** | **Peru** | **El Salvador** |
| --- | --- | --- | --- | --- | --- | --- | --- | --- | --- | --- | --- |
| **Existence of formal guidelines for RWE use** | No | No | No | Yes | No | No | No | Yes | No | No | No |
| **What uses indicated in the guideline?** |  |  |  | Adverse events |  |  |  | Introduction/context, adverse events, costs |  |  |  |
| **Uses RWE?** | No | Yes | Yes | No | Yes | No | Yes | Yes | Yes | Yes | No |
| **RWE used for what?** |  | Comp. Effectiveness, monitoring or training | Introduction/context, adverse events, costs, monitioring or training |  | Introduction/context, adverse events, disease burden, costs, monitoring or training |  | Other | Introduction/context, adverse events, costs, | Introduction/context, adverse events, disease burden, costs, comparative effectiveness, monitoring or training. | Introduction/context, adverse events, comparative effectiveness |  |
| **Evolution of RWE use in last 3 years** | No change | No change | Increase | No change | No change | Increase | No change | Increase | Increase | Increase | No change |
| **Evolution of the demand for RWE by stakeholders** | Increase | Increase | Increase | Increase | No change | Increase | Increase | Increase | Increase | Increase | No change |
| **Stakeholders requesting RWE in last 3 years** | Ministry of Health | Ministry of Health, technology producers, HTA agencies | Ministry of Health, private funders, technology producers, patients, professional societies, HTA agencies | Ministry of Health, private funders, technology producers, patients, professional societies, HTA agencies | Ministry of Health, private funders, technology producers, professional societies, HTA agencies | Ministry of Health, HTA agencies | Private funders, technology producers | Ministry of Health, patients, professional societies, other | Ministry of Health, technology producers, patients, professional societies | Private funders, technology producers, patients, professional societies | Ministry of Health, technology producers, HTA agencies, others |

| **Country** | **Uruguay** | **Chile** | **Paraguay** | **Costa Rica** | **Chile** | **Mexico** | **Argentina** | **Colombia** | **Brazil** | **Peru** | **El Salvador** |
| --- | --- | --- | --- | --- | --- | --- | --- | --- | --- | --- | --- |
| **Areas considered to be priority for increased RWE use** | Adverse events, disease burden estimations, costs, comparative effectiveness, monitoring or supervision. | Comparative effectiveness, creation of risk sharing agreements, monitoring or supervision. | Adverse events, costs, comparative effectiveness, monitoring or supervision. | Adverse events, disease burden estimations. | Costs, monitoring or supervision. | Adverse events, disease burden estimations, intervention costs, comparative effectiveness. | Comparative effectiveness. | Adverse events, costs, creation of risk sharing agreements. | Costs, comparative effectiveness, creation of risk sharing agreements, monitoring or supervision. | Adverse events, comparative effectiveness. | Adverse events, comparative effectiveness. |
| **Is RWE used for assessing comparative effectiveness?** | Not used | Not used | Sometimes used | Not used | Not used | Sometimes used | Not used | Always used | Sometimes used | Sometimes used | Not used |
| **Is a specific tool is used to assess the quality of RWE studies?** |  |  | No |  |  | No |  | No | Yes | No |  |
| **Name of the tool used** |  |  |  |  |  |  |  |  | ROBINS |  |  |
| **Conditions (diseases or technologies) for which RWE is most used for comparative effectiveness?** |  |  | High cost and orphan diseases |  |  | Orphan drugs |  | Orphan diseases, oncology, HIV | When RCTs are not sufficient for decision making | Devices and procedures |  |
| **Additional comments** |  |  | Promote capacity building courses for HTA assessors. |  |  |  |  | Improve quality of data and information systems |  |  | Updating HTA technical guides to include uses of RWE |
